# Reviewers in 2021

**DOI:** 10.1098/rspa.2022.0109

**Published:** 2022-03

**Authors:** Michael Lockwood

**Affiliations:** *Editor-in-Chief*

The Editors of *Proceedings of the Royal Society A* would like to thank all reviewers who contributed their time by refereeing manuscripts submitted throughout 2021. From previous feedback, we know that most reviewers value recognition of their efforts by the journal and through services such as Publons, which is integrated with *Proceedings A*.

To provide an opportunity for recognition, we list the names of all reviewers who have opted to be included. This article is made permanent and citable by its digital object identifier (DOI). We encourage you to quote your contribution in your applications for tenure and grants or other forms of research assessment. Where you have provided it, we have included your ORCID so your contribution can be unambiguously assigned to you.

On behalf of all the Editors and editorial office, I thank all of our reviewers past, present and future, and we look forward to your continued support of *Proceedings of the Royal Society A*.

Aaronson S

Abel M

Achcar JA

Adams H

Admal N

Afik Y https://orcid.org/0000-0001-8102-356X

Agarwal P https://orcid.org/0000-0001-7556-8942

Agliari A https://orcid.org/0000-0002-5230-9304

Agliari E

Aguilar Madera C

Altschul B https://orcid.org/0000-0002-3297-7815

Alves L https://orcid.org/0000-0001-6204-5552

Amabili M https://orcid.org/0000-0001-9340-4474

Anastopoulos C

Andersson L https://orcid.org/0000-0002-6364-7384

Andrews R

Anjos P https://orcid.org/0000-0002-3007-0531

Antipov Y https://orcid.org/0000-0002-2687-6081

Antipov YA

Arampatzis G

Arsioli B

Asllani M

Assier R https://orcid.org/0000-0001-9848-3482

Atchudan R

Avendano C

Azimi P

Babaee H https://orcid.org/0000-0002-6318-2265

Baddoo P https://orcid.org/0000-0002-8671-6952

Badri SH

Baek C

Bakke K https://orcid.org/0000-0002-9038-863X

Baltazar A

Bao T

Bardella L https://orcid.org/0000-0002-6042-0600

Barlow D

Barlow N

Barreto E

Basile I

Bassom A https://orcid.org/0000-0003-3275-7801

Batra R

Bazighifan O https://orcid.org/0000-0002-7251-9608

Beigi S

Bellis C

Belyaeva T

Berjamin H https://orcid.org/0000-0001-6637-8485

Bernard C

Bernard J-M

Bertagni M

Bhattacharjee S

Bhattacharya S

Bierbach D

Biggins J

Bigoni D https://orcid.org/0000-0001-5423-6033

Bilge AH

Blasone M

Blumenthal A

Bocquet M

Boge FJ

Bonder Y

Bora SN https://orcid.org/0000-0003-3242-1638

Bosia F https://orcid.org/0000-0002-2886-4519

brambilla e https://orcid.org/0000-0003-2515-528X

Brañas Garza P

Brassart L

Braverman A https://orcid.org/0000-0001-5127-9230

Breidenthal R https://orcid.org/0000-0002-5050-6267

Brianzoni S

Briz-Redón Á

Brun M https://orcid.org/0000-0002-4760-9062

Brunet P

Brunton S https://orcid.org/0000-0002-6565-5118

Buckley M

Bühler O

Bursten J

Bury T https://orcid.org/0000-0003-1595-9444

Busch T https://orcid.org/0000-0003-0535-2833

Bussey B

Caligiuri L

Camporeale C

Cao S

Capone F

Caravelli F https://orcid.org/0000-0001-7964-3030

Carboni B

Carletti T https://orcid.org/0000-0003-2596-4503

Carneiro da Cunha B

Carter B https://orcid.org/0000-0003-0469-1809

Casey J

Caso-Huerta M https://orcid.org/0000-0003-3367-1831

Castro L

Catlow R

Cattaneo M

Cavalcanti E https://orcid.org/0000-0001-9627-0520

Cavazzini G

Cegla F

Chakraborty S

Champneys A https://orcid.org/0000-0001-7772-3686

Chang DC

Chang W https://orcid.org/0000-0002-0735-0447

Chanyal BC

Chau JL

Chen G

Chen J

Chen P

Chen X https://orcid.org/0000-0002-9129-2197

Chen Y https://orcid.org/0000-0002-6008-6542

Cheng Y-T

Chernyavsky I https://orcid.org/0000-0003-0284-9318

Cheung SW https://orcid.org/0000-0002-8503-8395

Cho M

Chowdury A

Christou M https://orcid.org/0000-0002-6816-5448

Christov I https://orcid.org/0000-0001-8531-0531

Ciarletta M

Ciarletta P https://orcid.org/0000-0002-1011-5587

Cieśliński J https://orcid.org/0000-0003-1730-0950

Coelho PJ

Collins R

Colombo RM

Colorado E

Colquitt D https://orcid.org/0000-0001-5637-1626

Condurache D https://orcid.org/0000-0001-9287-8387

Copar S https://orcid.org/0000-0002-7566-0260

Copping A

Coste C

Coupier G https://orcid.org/0000-0001-5010-4148

Coutinho R

Cox A

Cox S

Crispino L

Cui Y

Czuba J

Dai M

Dalal PDC

d'angelo M

Davison M

Dayal K https://orcid.org/0000-0002-0516-3066

de Gosson M https://orcid.org/0000-0001-8721-1078

de León M

Deangelis D

Deb D

Dehghanpour H https://orcid.org/0000-0002-1434-3237

Dehmamy N

Delplace P

Demontis F

DeSimone A https://orcid.org/0000-0002-2632-3057

Devloo P https://orcid.org/0000-0002-8225-1107

Dhas B

Di Federico V

Di Molfetta G https://orcid.org/0000-0002-1261-7476

Dieks D

Dijksman J

Dimitri R

Dobie G

Domingos EF

Dong S-H https://orcid.org/0000-0002-0769-635X

Drazer G

Drinkwater BW https://orcid.org/0000-0002-8307-1175

Drossinos Y

Drozdov A

D'Souza K

Du J

Dunham E

Dunn D

Dutta R https://orcid.org/0000-0003-0195-2153

Dutta R

Egberts P

Elkouss D

Elze H-T

Ephremidze L https://orcid.org/0000-0002-1000-3841

Eriksson A

Escobar-Ruiz M

Eugster S https://orcid.org/0000-0002-4562-1287

Fairley I https://orcid.org/0000-0001-8562-7352

Fang H https://orcid.org/0000-0001-6691-0531

Farajpour Ouderji A

Favier B

Fedele L https://orcid.org/0000-0002-8459-0700

Feng B-F https://orcid.org/0000-0002-2529-897X

Feng ZC

Ferrara M

Fiziev P

Fontelos M https://orcid.org/0000-0003-4986-1752

Forbes L https://orcid.org/0000-0002-9135-3594

Fossati M https://orcid.org/0000-0002-1165-5825

Fragkoulidis G

Freire I https://orcid.org/0000-0003-0539-9674

Frigo M https://orcid.org/0000-0002-9365-8017

Fröhlich F

Fu F https://orcid.org/0000-0001-8252-1990

Fu H https://orcid.org/0000-0002-5400-3042

Fujii G

Galatola P

Gamboa PV

Gameros Madrigal A https://orcid.org/0000-0002-1508-8434

Gao Y

Gardner S

Gayen R https://orcid.org/0000-0002-7204-6951

Germain G

Germano G

Ghil M https://orcid.org/0000-0001-5177-7133

Gholipour M https://orcid.org/0000-0001-6290-9461

Giansiracusa J

Gilbert A

Girish TE https://orcid.org/0000-0002-6942-2434

Girolami M

Gleeson J https://orcid.org/0000-0003-3410-2817

Gnann M

Goddard J

Godoy-Diana R https://orcid.org/0000-0001-9561-2699

Gorecki W

Gracia-Lázaro C https://orcid.org/0000-0002-9769-8796

Gradoni G https://orcid.org/0000-0001-8321-6883

Graziani F

Green C https://orcid.org/0000-0001-5181-4037

Griffiths D https://orcid.org/0000-0002-4406-4781

Griffiths I https://orcid.org/0000-0001-6882-7977

Grigorieva E

Grima R https://orcid.org/0000-0002-1266-8169

Grmela M

Grobler J

Groh R https://orcid.org/0000-0001-5031-7493

Gu E-G

Guevara Vasquez F https://orcid.org/0000-0001-8746-3582

Guo X

Gupta A

Gurao N

Hackl L https://orcid.org/0000-0002-4172-0317

Hajek B

Hale J

Hall C

Halliwell J

Harikrishnan AR

Harko T

Hatsuda Y

Haug E https://orcid.org/0000-0001-5712-6091

Haw M

Hazel A

Hazra A https://orcid.org/0000-0002-8658-9412

He J https://orcid.org/0000-0002-2068-1849

Hébert R https://orcid.org/0000-0002-9869-4658

Hendi SH

Henghes B

Herbert C

Herrera F

Hessler M https://orcid.org/0000-0003-0529-7926

Heydari MH

Hinze T

Hochberg D

Hohermuth B

Hollingsworth J

Hone A https://orcid.org/0000-0001-9780-7369

Hong W-C https://orcid.org/0000-0002-3001-2921

Horikis T https://orcid.org/0000-0003-0731-6107

Hu G

Hu L

Huang R

Huang Y

Huang YM

Hulshoff S

Hurley R http://orcid.org/0000-0003-1652-635X

Huthwaite P https://orcid.org/0000-0003-4433-4056

Hwang G

Iacopini I

Iizuka H

Ioannou P

Iquiapaza R https://orcid.org/0000-0003-1657-2823

Irvine-Fynn T https://orcid.org/0000-0003-3157-6646

Isham V

Ivanov R https://orcid.org/0000-0003-1008-8272

Jackson R

Jacobs D

Jaiman R

Jansen M https://orcid.org/0000-0003-4753-3173

Jansson P https://orcid.org/0000-0002-8832-8806

Jawed M

Jeynes C

Jóźwiak B

Jung P

Juniper M

Kabir KM https://orcid.org/0000-0003-0249-5417

Kadic M

Kalogirou A

Kapaklis V

Karafyllidis I

Karr J-P https://orcid.org/0000-0003-2082-0914

Karsten R

Katriel J

Kattnig D

Katz R

Kavvas L

Keeler R

Kent A https://orcid.org/0000-0003-2624-3687

Kerner R

Kessler D

Khalique C

Khiêm VN

Kholmetskii A https://orcid.org/0000-0002-5182-315X

Khonsari M https://orcid.org/0000-0003-3192-8891

Kidambi N

Kiessling M

Kim S-C

Kirillov O

Kissinger A https://orcid.org/0000-0002-6090-9684

Klinkhamer FR

Kondrashov D

Konispoliatis D

Konstantinidis D

Kontis K

Kopeikin S https://orcid.org/0000-0002-4866-1532

Koutsawa Y https://orcid.org/0000-0002-9016-1531

Kovács F

Kozuka Y

Kraniotis G

Křen P

Kroger M https://orcid.org/0000-0003-1402-6714

Kumar S

Kwon O

Lai F

Laing C

Lakin M https://orcid.org/0000-0002-8516-4789

Landel J https://orcid.org/0000-0003-3159-8749

Landsman K

Langenberg S https://orcid.org/0000-0001-5817-5469

Larsson R

Laude V

Lawley S

Lazar N

Le Clainche S

Lee CH

Lekner J https://orcid.org/0000-0003-4675-9242

Lellouch A

Lenells J https://orcid.org/0000-0001-6191-7769

Leonetti L https://orcid.org/0000-0001-7182-2149

Lermusiaux P https://orcid.org/0000-0002-1869-3883

Lestringant C https://orcid.org/0000-0002-6929-4655

Levene M

Lewis A

Li B https://orcid.org/0000-0002-3792-2469

Li J

Li K https://orcid.org/0000-0002-6699-6264

Li Q

Li R

Li W

Liao S https://orcid.org/0000-0002-2372-9502

Lim CW https://orcid.org/0000-0003-1030-9063

Lindsay A

Ling L https://orcid.org/0000-0002-3051-4366

Lior I

Liu D

Liu J https://orcid.org/0000-0001-9944-4493

Liu K

Liu M

Liu S

Liu T https://orcid.org/0000-0003-2713-3674

Liu X https://orcid.org/0000-0003-0235-1894

Liu Z

Llewellyn Smith S https://orcid.org/0000-0002-1419-6505

Lo W-C https://orcid.org/0000-0001-7771-5213

Longbin T

Longo IP https://orcid.org/0000-0003-1820-4274

Longo S

Lopez J

Lopez-Garcia M https://orcid.org/0000-0003-3833-8595

Lorenzini M https://orcid.org/0000-0002-4585-8575

Love P

Lubarda MV

Lucarini V https://orcid.org/0000-0001-9392-1471

Lucia U https://orcid.org/0000-0002-3123-2133

Luongo A

Lushnikov P https://orcid.org/0000-0001-6988-5044

Ma J

Ma W-X

Maccone L

Macias-Diaz J

Maclaren O

Madzvamuse A https://orcid.org/0000-0002-9511-8903

Mai Q

Majumdar A https://orcid.org/0000-0003-4802-6720

Malla R

Malomed B https://orcid.org/0000-0001-5323-1847

Mandolesi A https://orcid.org/0000-0002-5329-7034

Manfredi P

Mann R https://orcid.org/0000-0003-0701-1274

Mari R

Mariani VC

Maroulas V

Martini E

Mascia C

Mason HB

Mattheakis M

Mattingly D

Matzkin A

Mazdziarz M

Mazumder B https://orcid.org/0000-0002-8891-1559

McCall M

McCarthy A

McEvoy E

McKinley S https://orcid.org/0000-0001-9434-9163

Mendoza S

Mentrelli A

Mériaux C https://orcid.org/0000-0003-1683-8572

Micheletti A

Michelitsch T https://orcid.org/0000-0001-7955-6666

Mihai A https://orcid.org/0000-0003-0863-3729

Minguzzi E

Misbah C

Misseroni D

Mitsotakis D

Molas M

Mollon G https://orcid.org/0000-0001-5486-8129

Montanher T

Moulton D

Mück W

Muruganandam P https://orcid.org/0000-0002-3246-3566

Nakajima K

Nakata Y

Nan T

Natario J https://orcid.org/0000-0003-0885-9867

Nath G

Navon IM

Nelias D https://orcid.org/0000-0002-1080-5238

Neukirch S

Nguyen VH https://orcid.org/0000-0003-1959-9087

Nicks R

Nieto JJ

Nieves M https://orcid.org/0000-0003-4616-4548

Nishijima M

Noventa S

Oberst S https://orcid.org/0000-0002-1388-2749

Okon E

Ostoja-Starzewski M https://orcid.org/0000-0002-3493-363X

Pagani A

Palffy-Muhoray P https://orcid.org/0000-0002-9685-5489

Pan J

Pan S https://orcid.org/0000-0002-2462-362X

Panagiotou E https://orcid.org/0000-0002-1655-6447

Panou G

Papangelo A https://orcid.org/0000-0002-0214-904X

Pappa A

Paprota M https://orcid.org/0000-0001-6642-2589

Parker D

Pasini D https://orcid.org/0000-0002-3021-7118

Pasquier P

Pažanin I

Pelinovsky D https://orcid.org/0000-0001-5812-440X

Pence T

Peng W

Peng Z

Peotta S

Perčić M https://orcid.org/0000-0002-5873-0263

Perdikaris P https://orcid.org/0000-0002-2816-3229

Petri G https://orcid.org/0000-0003-1847-5031

Petrov P

Pezzulla M https://orcid.org/0000-0002-3165-8011

Phang C

Piccolroaz A

Pillai D https://orcid.org/0000-0002-7607-7923

Pinheiro H

Pinheiro M

Pires L

Placidi L https://orcid.org/0000-0002-1461-3997

Plávala M https://orcid.org/0000-0002-3597-2702

Podgornik R

Policicchio A

Popovych R https://orcid.org/0000-0003-2403-1525

Portegies Zwart S

Portone T

Porubov A

Potts P

Pourahmadian F

Prakash BA

Prendergast L

Prikazchikov D

Protas B https://orcid.org/0000-0003-3935-3148

Pugliese A

Pujara N https://orcid.org/0000-0002-0274-4527

Puttanna V https://orcid.org/0000-0001-6652-1325

Qiao Z https://orcid.org/0000-0002-5578-6181

Qin Z

Qu Y

Rabault J

Radi E https://orcid.org/0000-0002-7410-3008

Raffaelli M https://orcid.org/0000-0002-4908-196X

Rai D

Raines ZM

Rajchakit G https://orcid.org/0000-0001-6053-6219

Ralph T

Ramos R

Ran A

Raposo A https://orcid.org/0000-0002-1263-599X

Rath B https://orcid.org/0000-0001-7512-1702

Ratnasingam S

Razani A

Razzaghi M

Ribodetti A

Ricca R https://orcid.org/0000-0002-7304-4042

Richards F

Ringbauer M

Robert C https://orcid.org/0000-0001-6635-3261

Rohlfs W

Rokoš O

Romano V

Romero D https://orcid.org/0000-0002-3603-7361

Rosa M

Rosales-Zárate L

Rose LRF

Rosetto V

Rosti ME https://orcid.org/0000-0002-9004-2292

Rovelli C https://orcid.org/0000-0003-1724-9737

Royston T

Ruschhaupt A

Russo L

Saalwächter K

Saccomandi G https://orcid.org/0000-0001-7987-8892

Sadoulet-Reboul E

Sahoo PK

Sahu KC

Sakajo T https://orcid.org/0000-0002-4290-0942

Sakalli I

Sambas ‪

Sanchez Garcia R

Sandstede B https://orcid.org/0000-0002-5432-1235

Sanduk M

Sanz Á

Saridakis EN

Sassa S https://orcid.org/0000-0002-0269-200X

Sauret A

Sautbekov S

Sazhin S

Scapellato A

Schehr G

Schnitzer O https://orcid.org/0000-0002-5608-2311

Schüler M

Schulze K

Schweizer B

Sciacca M

Seadawy A https://orcid.org/0000-0002-7412-4773

Sebens C

Sen U

Seroussi H

Serrano-Aroca Á

Severino F

Shailesh S

Sharma B https://orcid.org/0000-0002-4968-9203

Shemer L

Shen J

Shepelsky D

Sherwood J https://orcid.org/0000-0002-3231-2688

Shittu S

Sieber J https://orcid.org/0000-0002-9558-1324

Siettos C

Siewe N

Sikora P

Silva CJ

Simley E

Singer A

Skokos C

Sledge D

Smalyukh I

Smith D

Smyth N

Snoeijer J https://orcid.org/0000-0001-6842-3024

Sobac B

Sobczak S

Sobhani H

Soda B

Sofiyev A

Soldatos K https://orcid.org/0000-0003-3323-5220

Son S https://orcid.org/0000-0002-2819-5140

Song M

Spagat M

Speekenbrink M

Spigarelli S https://orcid.org/0000-0003-1429-0224

Spitkovsky I https://orcid.org/0000-0002-1411-3036

Spivak D

Srinivasa A

Staat M https://orcid.org/0000-0003-4363-6570

Stachura E https://orcid.org/0000-0002-5105-2914

Stagonas D

Steinbock O

Stephen R

Stewart P https://orcid.org/0000-0002-0971-8057

Stolle J

Stovas A

Struchtrup H https://orcid.org/0000-0002-4986-0728

Struyve W

Strzałkowski J https://orcid.org/0000-0001-7001-9303

Su Y https://orcid.org/0000-0002-5583-4251

Sudbery A https://orcid.org/0000-0002-8408-9510

Susanto H

Szolnoki A

Tabak AF

Tabatabaeipour M https://orcid.org/0000-0002-9355-3813

Taber KS

Taffetani M https://orcid.org/0000-0001-9587-156X

Tai Y-C https://orcid.org/0000-0001-5925-8207

Tambača J

Tang E

Tang J https://orcid.org/0000-0002-4963-1028

Tanizawa T

Tanner G

Terry C

Thampi S

Tian S-F https://orcid.org/0000-0003-0888-777X

Tibullo V https://orcid.org/0000-0001-9861-1210

Tiso P

Tlusty T https://orcid.org/0000-0002-9662-3725

Toffoli A https://orcid.org/0000-0003-3112-6856

Tohidi E

Tomassetti G

Topp C

Tornabene F

Toth L

Transtrum M

Trentadue F

Trinh P https://orcid.org/0000-0003-3227-1844

Tsaneva-Atanasova K

Tsuzuki MSG

Tuffen H

Tura J

TURCO E https://orcid.org/0000-0002-8263-7034

Tyranowski T https://orcid.org/0000-0002-9078-5950

Tzavaras A

Ur Rehman N

Vacchini B

Vadasz P https://orcid.org/0000-0003-4996-9341

Vaidman L https://orcid.org/0000-0001-5907-7553

Valagiannopoulos C

Valentini A

Valido AA

van de Weygaert R

van den Bremer T

van der Heijden G https://orcid.org/0000-0002-0720-5469

Van Gorder R https://orcid.org/0000-0002-8506-3961

Van hoorickx C https://orcid.org/0000-0002-9671-5558

Ván P https://orcid.org/0000-0002-9396-4073

Vasan V

Veeresha P https://orcid.org/0000-0002-4468-3048

Vellender A

Vendeville A https://orcid.org/0000-0002-9044-8348

Venkataramani S

Verhulst F

Vesga-Ramirez A

Vidal F

Vilarrasa V

Vitória R

Vo T https://orcid.org/0000-0003-4599-0215

Volkmer H

Waegell M

Wainwright J

Wake GC https://orcid.org/0000-0002-2749-291X

Walker M https://orcid.org/0000-0001-9910-2506

Wallace D

Wang B

Wang D-S

Wang L

Wang Liqiu http://orcid.org/0000-0002-6514-4211

Wang Y

Wang Z

Watanabe T https://orcid.org/0000-0003-3203-3504

Watson S

Weber S

Weigert S

Wen J

Wen X-Y

Wilcox P https://orcid.org/0000-0002-8569-8975

Winter M

Wiseman H

Wu-Ming L

Wunderling N

Xiang Z

Xiao J-J

Xu F https://orcid.org/0000-0003-3910-5398

Xu J

Yadav D https://orcid.org/0000-0001-6051-479X

Yahalom A https://orcid.org/0000-0001-6679-3320

Yamamoto M

Yamauchi Y

Yan J

Yan S

Yan W

Yang H https://orcid.org/0000-0002-3905-436X

Yang H

Yang S https://orcid.org/0000-0002-7886-8446

Yang X https://orcid.org/0000-0003-0882-2650

Yavuz M https://orcid.org/0000-0002-3966-6518

Yeh H

Yi J

Yin J

Ying Y https://orcid.org/0000-0001-8216-0910

Young DL

Yu Y

Yuan T

Yüzbaşı Ş https://orcid.org/0000-0002-5838-7063

Zanella M https://orcid.org/0000-0001-8456-5866

Zhang J https://orcid.org/0000-0002-7576-6110

Zhang T

Zhao F https://orcid.org/0000-0003-0515-6808

Zhao L

Zhou S https://orcid.org/0000-0001-9658-9195

Zhu Q

Zhu Z-N https://orcid.org/0000-0001-6369-7552

Zingales M https://orcid.org/0000-0001-9093-9529

Zou W https://orcid.org/0000-0001-8442-377X

Zou X

Zulli D

Zurlo G

